# Tumor necrosis factor-α coordinates the macrophage innate immune response to *Histoplasma capsulatum*

**DOI:** 10.1128/iai.00302-26

**Published:** 2026-09-29

**Authors:** Bradford L. Crowther, William Buesing, Ty Troutman, George S. Deepe

**Affiliations:** 1 Division of Infectious Diseases, Department of Medicine, University of Cincinnati College of Medicine 12303, Cincinnati, Ohio, USA; 2 Department of Molecular and Cellular Biosciences, University of Cincinnati College of Medicine 12303, Cincinnati, Ohio, USA; 3 Division of Allergy and Immunology, Department of Pediatrics, Cincinnati Children’s Hospital Medical Center, Cincinnati, Ohio, USA; University of Illinois Chicago, Chicago, Illinois, USA

**Keywords:** lung, *Histoplasma*, macrophages, tumor necrosis factor

## Abstract

*Histoplasma capsulatum* is a fungal pathogen that thrives in macrophages until activated by interferon-gamma (IFN-γ). Tumor necrosis factor-α (TNF-α) is required for control of infection during adaptive immunity but does not directly activate macrophages to eliminate fungal elements. Collapse of immunity associated with TNF-α blockade is not accompanied by depression of IFN-γ. To determine if TNF-α antagonism impairs fungal clearance prior to adaptive immunity, we infected mice given anti-TNF-α and quantified lung *H. capsulatum* up to day 3 of infection. Anti-TNF-α elevated the number of organisms in the lungs by day 3. Neutralization of IFN-γ manifested a similar effect. Since neutralization of both cytokines impaired innate immunity, we hypothesized that the action of IFN-γ required TNF-α. IFN-γ-stimulated macrophages expressed fungicidal activity *in vitro*, and TNF-α neutralization or its congenital deficiency in macrophages reversed this effect. Blockade of TNF-α slightly dampened M1 response but did not promote M2. TNF-α was not required for IFN-γ to shift the metabolic capacity of macrophages. We investigated the production of nitric oxide, which is a mediator of anti-*H*. *capsulatum* activity by macrophages. In the absence of TNF-α, there was no difference when compared to IFN-γ stimulation alone. Lung macrophages harbored the highest infection burden in anti-TNF-α recipients. Though IFN-γ receptor 1 was modestly downregulated by anti-TNF-α blockade, this treatment downregulated pSTAT1 at day 2 of infection. TNF-α is required for optimal IFN-γ signaling and effector function of cytokine-stimulated macrophages. When TNF-α is absent, macrophages are insufficiently activated and fail to halt intracellular growth of yeast cells.

## INTRODUCTION

Tumor necrosis factor-α (TNF-α) is a multifunctional cytokine involved in the establishment of inflammatory processes, cell proliferation, and survival (1, 2). Antimicrobial activities associated with this cytokine are achieved by stimulating inflammatory responses to invading pathogens or by provoking the production of interferon-gamma (IFN-γ) from innate lymphocytes (3–6). TNF-α is a critically important component of host defenses to many pathogens, including the dimorphic fungal pathogen *Histoplasma capsulatum*, the causative agent of histoplasmosis (7). However, exaggerated generation of TNF-α is detrimental to health, as observed in multiple inflammatory conditions such as rheumatoid arthritis (8, 9). In the clinical arena, TNF-α antagonists ameliorate the damaging inflammatory conditions associated with overproduction of the cytokine, but these therapeutics pose a major risk factor for the development of several diseases, including fungal infections (9–11). TNF-α blockade disables protective immunity against *H. capsulatum* in humans and mice, leading to a failure to control infection (12–15).

Despite the necessity of TNF-α as a master regulator of protective immunity and proinflammatory stimuli, this cytokine does not directly activate antimicrobial defenses of the macrophage to inhibit growth of *H. capsulatum* (16). Like TNF-α, IFN-γ is required for recovery from *H. capsulatum* infection (14, 17). These two soluble mediators synergize at the level of their associated signaling pathway components in a careful balancing act to produce a proinflammatory response (18). Previous studies from our laboratory demonstrated that neutralization of TNF-α causes the collapse of immunity to *H. capsulatum*, despite generation of IFN-γ in lungs similar to if not slightly more than that found in controls. Why IFN-γ fails to exert antifungal activity when TNF-α is blocked remains enigmatic. Although TNF-α cooperates with IFN-γ to coordinate activation of macrophages, the complete lack of protective immunity when TNF-α is absent but IFN-γ is present suggests that the interaction between these two cytokines goes beyond synergy. How these two cytokines act in concert to coordinate antimicrobial defenses against *H. capsulatum* during the innate immune response remains unclear.

Here, we show that TNF-α and IFN-γ are independently necessary to limit infection during the innate phase of immunity *in vivo* (≤7 days). We establish that *H. capsulatum*-infected macrophages exposed to IFN-γ require endogenous TNF-α to exert intracellular killing. Although IFN-γ-induced rewiring of macrophage metabolism is a key element in the antimicrobial actions of this cytokine, we found that blockade of TNF-α did not alter glycolysis or mitochondrial respiration. While metabolic changes induced by IFN-γ activation are independent of TNF-α, we observed no difference in the presence of reactive nitrogen intermediates in these conditions. Investigating lung cell populations, we discovered that macrophages were the most prevalent infected populations in the absence of TNF-α. TNF-α blockade caused no perturbations in IFN-γ receptors but did decrease signaling at the pSTAT1 level, leading to dampened interferon-stimulated gene expression. These findings suggest that macrophages are responsive to IFN-γ in the absence of TNF-α but are not sufficiently activated to control *H. capsulatum* replication without TNF-α as a critical comediator.

## RESULTS

### TNF-α and IFN-γ are required for early control of *H. capsulatum* pulmonary infection

**Fig 1:**
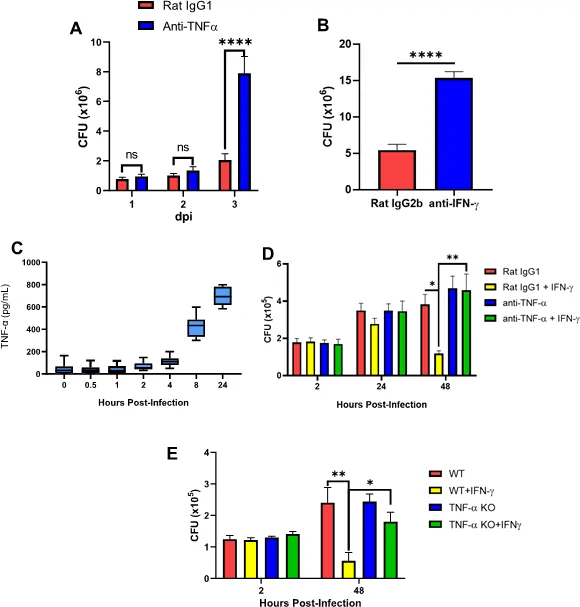
TNF-α is required for activation of macrophages by IFN-γ to control *H. capsulatum* infection. (**A**) CFUs from lungs 1, 2, and 3 days post-infection from *H. capsulatum*-infected mice treated with either anti-TNF-α (blue) or rat IgG1 isotype control antibody (red). (**B**) CFUs from lungs 3 days post-infection from *H. capsulatum*-infected mice treated with either anti-IFN-γ or rat IgG1 isotype control antibody. Data are mean ± SEM (*n* = 8, ****P < 0.0001). (**C**) TNF-α secretion in BMDMs over time up to 24 hours post-infection (*n* = 12). (**D**) CFUs from BMDMs at 2, 24, and 48 hours post-infection. Rat IgG1, yellow; rat IgG1 + IFN-γ, red; anti-TNF-α, blue; and anti-TNF-α + IFN-γ, green. Data are mean ± SEM (*n* = 6, *P = 0.0003, **P < 0.0001). (**E**) Comparison of TNF-α knockout (KO) BMDMs to WT at 2 and 48 hours post-infection infected with *H. capsulatum* and treated with IFN-γ. Data are means ± 1 standard error of the mean (*n* = 7; WT in red, WT + IFN-γ in yellow, TNF-α KO in blue, and TNF-α KO + IFN-γ in green).

Our lab has previously shown that TNF-α and IFN-γ are independently required for recovery from *H. capsulatum* infection in mice. Those studies focused largely on the period of infection regulated by adaptive immunity. The influence of these cytokines on the innate immune response to *H. capsulatum* has yet to be fully investigated (14, 17). To determine the impact of TNF-α ablation in early infection, mice were injected with anti-TNF-α monoclonal antibody (mAb) or isotype control antibody before infection. At 1, 2, and 3 days post-infection (dpi), mice were euthanized, and their lungs were cultured for *H. capsulatum*. While there was a progressive increase in lung colony-forming units (CFUs) recovered from anti-TNF-α-treated mice compared to infected controls at 1 and 2 dpi, by day 3, the numbers of CFUs in the lungs of anti-TNF-α-treated mice greatly exceeded those of controls (Fig. 1A, *P* < 0.0001).

Prior literature has shown IFN-γ to be equally as important as TNF-α for recovery from infection (14, 17). To compare our findings on the importance of TNF-α, we performed a similar experiment in mice treated with anti-IFN-γ mAb or control antibody. We observed an increase in *H. capsulatum* CFU recovered from anti-IFN-γ-treated mice at 3 dpi compared to controls, a pattern comparable to our finding with anti-TNF-α in our previous experiment (Fig. 1B, *P* < 0.0001).

### TNF-α is required for IFN-γ-mediated control of *H. capsulatum* infection

Based on our *in vivo* observations, we investigated whether TNF-α was required for IFN-γ to induce fungicidal activity in macrophages. Since TNF-α blockade did not alter IFN-γ generation in the lungs at 3 dpi, this prompted an inquiry as to whether TNF-α was required for activity of IFN-γ (14). Before performing that experiment, we examined the kinetics of TNF-α generation by bone marrow-derived macrophages (BMDMs) exposed to *H. capsulatum*. BMDMs began to secrete TNF-α at 2 hours post*-H. capsulatum* infection and continued to produce the cytokine up to 24 hours post-infection (Fig. 1C). We assessed the viability of BMDMs at 24 hours post-infection and found that viability was maintained compared to the uninfected control (data not shown). Next, we charted the course of infection in BMDMs at 2, 24, and 48 hours post-infection to replicate *in vitro* what we observed *in vivo*. We infected BMDMs with *H. capsulatum* yeasts and exposed infected cells to rat IgG1, anti-TNF-α, and/or IFN-γ at the time of infection. At 2, 24, and 48 hours post-infection, we assessed the fungal burden in BMDMs. At 48 hours post-infection, IFN-γ induced fungicidal activity in control macrophages; in contrast, IFN-γ did not induce killing of *H. capsulatum* when TNF-α was blocked (Fig. 1D). To complement these findings, we conducted a similar experiment using TNF-α^−/−^ macrophages and observed the same effect (Fig. 1E). These observations are consistent with prior studies that demonstrate TNF-α and IFN-γ are independently required for protective immunity against *H. capsulatum*, and rather than regulating IFN-γ production, TNF-α functions in early macrophage-derived licensing that enables optimal IFN-γ-mediated immunity to intracellular pathogens (14, 19).

### Differential expression of M1- and M2-associated genes during *H. capsulatum* infection in the absence of TNF-α

Our findings that IFN-γ is dependent on TNF-α to stimulate BMDMs to kill *H. capsulatum* spurred us to investigate TNF-α-dependent behaviors of macrophages during infection. We began by asking if the activation state of IFN-γ-stimulated macrophages relies on the presence of TNF-α. In this regard, TNF-α suppresses M2 gene expression in tumor-associated macrophages (20). Therefore, we explored the possibility that TNF-α blockade shifts a macrophage to an alternatively activated state in the presence or absence of IFN-γ. M2 macrophages are permissive to the intracellular growth of *H. capsulatum* and fail to respond to IFN-γ (21).

**Fig 2:**
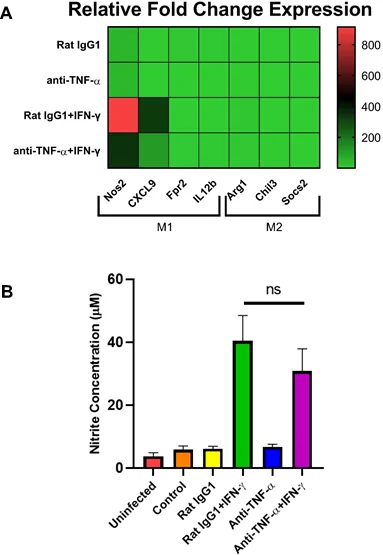
TNF-α is required for activation of macrophages by IFN-γ to control *H. capsulatum* infection but not for nitrite production. (**A**) Heatmap of selected M1- and M2-associated cell surface marker expression from samples retrieved at 24 hours post-infection measured by RT-PCR. M1 markers include *Nos2*, *CXCL9*, *Fpr2*, and *IL12b*. M2 markers include *Arg1*, *Chil3*, and *Socs2*. Values are relative fold changes in gene expression normalized from an uninfected control group (*n* = 4). (**B**) Nitrite detected in BMDM supernatants at 24 hours post-infection (*n* = 4, non-significant, ^ns^*P* = 0.4081).

We performed reverse transcription quantitative PCR on BMDMs of the same groups as in Fig. 1C, comparing all samples to an uninfected control at 24 hours post-infection to assess for expression of a selected group of M1- and M2-associated genes. Of the expression profiles evaluated, M1-associated genes showed increased expression in macrophages treated with IFN-γ, while M2-associated gene expression in macrophages was not significantly altered from that of a resting, uninfected macrophage (Fig. 2A). We observed a significant difference in expression of *Nos2*, an M1-associated marker, at 24 hours post-infection when comparing IFN-γ-treated groups with and without anti-TNF-α (Fig. 2A, *P* = 0.045).

### Production of nitric oxide intermediates is unaltered in absence of TNF-α

To complement our finding that *Nos2* expression was decreased in the anti-TNF-α + IFN-γ group, we questioned whether this change was associated with a decrease in nitric oxide (NO). Production of reactive nitrogen species (RNS) has been well documented in the context of *H. capsulatum* infection as a vital mechanism for host defense, but this has not been shown in the absence of TNF-α (22). The addition of IFN-γ increased the production of the NO intermediate, nitrite, independent of TNF-α (Fig. 2B). This finding shows that *Nos2* expression did not correlate with available nitrite for RNS production.

### Alterations in macrophage metabolism from IFN-γ stimulation are independent of TNF-α

**Fig 3:**
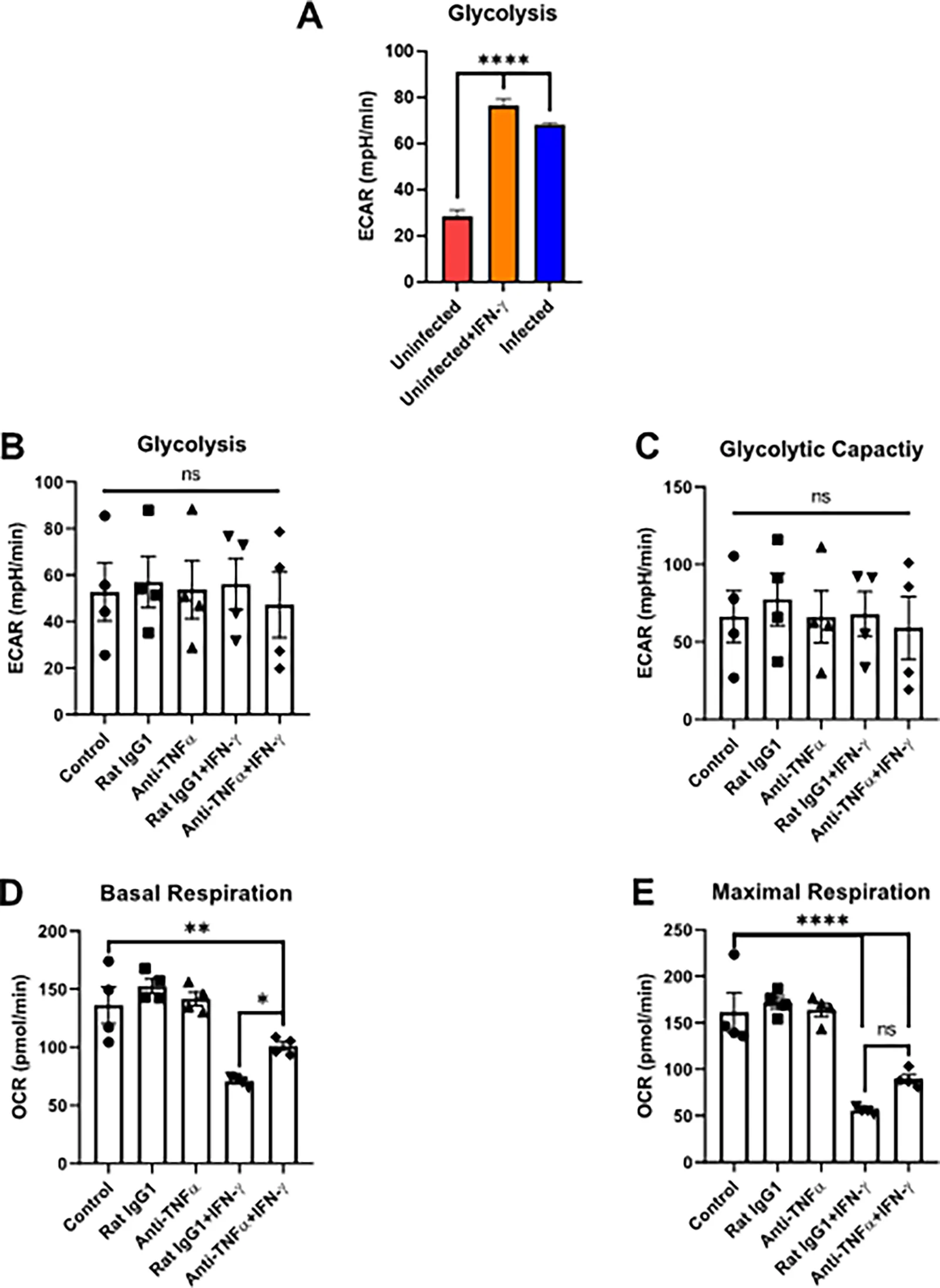
IFN-γ and TNF-α blockade does not alter glycolytic capacity but does lower mitochondrial respiration in *H. capsulatum* infection of BMDMs. (**A**) Glycolysis of cells stimulated with IFN-γ or infection measured as extracellular acidification rate (ECAR; *n* = 3, ****P < 0.0001), compiled by Seahorse XF Analyzer. (**B**) Glycolysis and (**C**) glycolytic capacity in *H. capsulatum*-infected macrophages treated with IFN-γ and anti-TNF-α at 24 hours post-infection (ECAR, *n* = 4). (**D**) Basal respiration and (**E**) maximal respiration in *H. capsulatum*-infected macrophages treated with IFN-γ and anti-TNF-α at 24 hours post-infection, compiled by Seahorse XF Analyzer and measured by oxygen consumption rate (OCR; *n* = 4, *P = 0.0242, **P = 0.0010, ****P < 0.0001, ns, non-significant, *P* > 0.05).

IFN-γ stimulation profoundly alters the metabolic profile of macrophages in a way that promotes proinflammatory responses (23). Macrophages deficient in HIF-1α, a key transcriptional regulator of the metabolic activity of these phagocytes, are unresponsive to IFN-γ because of elevated generation of interleukin-10 (22, 24). Since IFN-γ induces an M1 phenotype in the absence of TNF-α, we investigated if a deficiency of TNF-α altered the metabolic capacity of IFN-γ-stimulated macrophages. Using the extracellular flux assay, while glycolytic capacity was altered by both infection and IFN-γ separately, the profile was unchanged by the absence of TNF-α (Fig. 3A through C). Despite IFN-γ decreasing the basal respiration of macrophages infected with *H. capsulatum*, the effect on maximal mitochondrial respiratory capacity was partially independent of TNF-α (Fig. 3D and E).

### Macrophages and monocytes are the prime targets for *H. capsulatum* in early infection in absence of TNF-α

Two distinct resident macrophage populations exist in the lung: alveolar macrophages (AMs) and interstitial macrophages (IMs). We hypothesized that if *H. capsulatum* is primarily an infection of macrophages, one of these subpopulations should bear the burden of a higher rate of infection when TNF-α is absent. Inflammatory monocytes were included in this analysis, as these immune cells are infected with *H. capsulatum* (Fig. S1).

**Fig 4:**
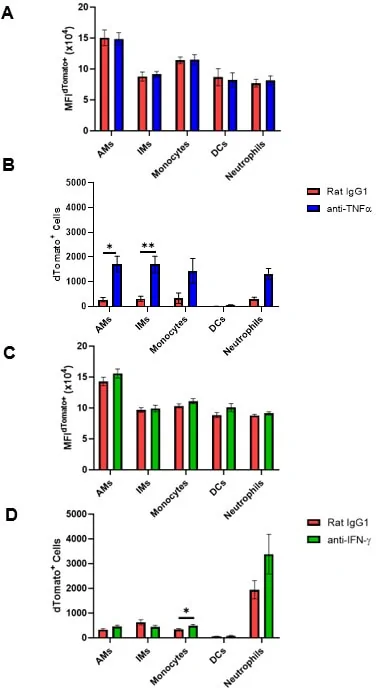
Analysis of lung leukocyte populations by flow cytometry. (**A**) MFI and (**B**) cell counts of tdTomato-expressing *H. capsulatum*-infected cells in control and anti-TNF-α antibody treatments (*n* = 12, *P = 0.0361, **P = 0.0203). (**C**) MFI and (**D**) cell counts of *H. capsulatum*-infected cells in control and anti-IFN-γ antibody treatments (*n* = 11, *P = 0.0376). Cell populations include AMs, defined as CD45^+^ F4/80^+^ CD64^+^ CD11b^−^ CD11c^+^ SiglecF^+^; IMs, defined as CD45^+^ F4/80^+^ CD64^+^ CD11b^+^ CD11c^+^ SiglecF^−^ MHCII^+^ CX_3_CR_1_^+^; monocytes, defined as CD45^+^ F4/80^+^ CD64^+^ CD11b^+^ CD11c^+^ SiglecF^−^ MHCII^−^ CD64^+^; DCs, defined as CD45^+^ F4/80^−^ CD11b^+/−^ CD11c^+^ CD64^−^ MHCII^+^; and neutrophils, defined as CD45^+^ F4/80^−^ CD11b^+^ CD11c^−^ CD64^−^ MHCII^−^.

Based on the median fluorescence intensities (MFIs) of dTomato^+^ *H. capsulatum*, the proportion of infection was similar between groups for each cell population analyzed (Fig. 4A). In the absence of TNF-α, AMs and IMs were observed to have a significantly increased number of infected cells compared to controls, while we observed no significant differences in monocytes, DCs, and neutrophils (Fig. 4B). Since neutralization of IFN-γ also disrupts innate immunity, we investigated whether blockade of this cytokine induced a similar profile to TNF-α ablation. While the dTomato^+^ MFI between groups and cell types remained consistent, the only statistically significant increase in infected cells was observed in monocytes (Fig. 4C and D). There was an insignificant increase (*P* > 0.05) in infected neutrophils. Although TNF-α or IFN-γ blockade similarly dampened innate immunity, the distribution of yeast cells in phagocytes differed substantially. These findings align with studies that demonstrate TNF-α and IFN-γ exert nonredundant roles in host defense against *H. capsulatum*. These data suggest that TNF-α is required to restrict fungal replication within resident lung macrophage populations (AMs and IMs), whereas IFN-γ appears to exert broader effects on recruited phagocytes (14, 19).

### Alterations in IFN-γ receptors during *H. capsulatum* infections are independent of TNF-α

**Fig 5:**
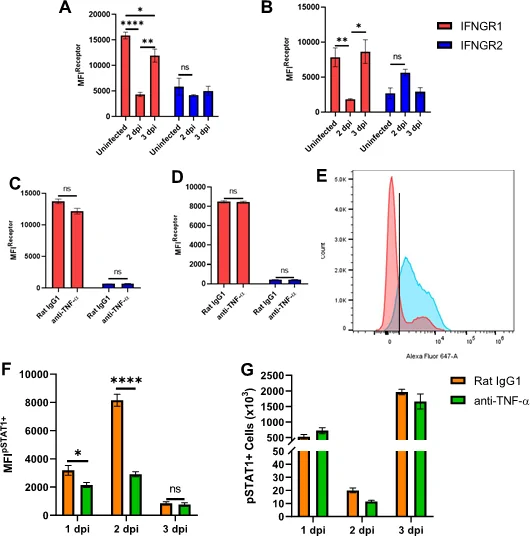
IFNGR expression on lung cell surfaces and intracellular pSTAT1 dynamics. IFNGR1 but not IFNGR2 is downregulated at 2 dpi in (**A**) CD45^+^ CD64^+^ (*P = 0.0061, **P = 0.0036, ****P < 0.0001), and (**B**) CD45^+^ CD64^−^ (*P = 0.0203, **P = 0.0178) cells compared to uninfected cells, then restored at 3 dpi. Data are means ± SEM (*n* = 6–8). In infected (**C**) CD45^+^ CD64^+^ and (**D**) CD45^+^ CD64^−^ cells treated with anti-TNF-α and a comparable isotype control antibody, there is no alteration in surface receptor content at 3 dpi (*n* = 6–8). (**E**) Representative histogram back gate of cells 24 hours post-infection displays the difference between isotype control (red) and pSTAT1 (blue) stains on the same fluor. The line indicates where pSTAT1^+^ is determined. (**F**) MFI and (**G**) absolute cell numbers of pSTAT1 between Rat IgG1 and anti-TNF-α-treated groups at 1, 2, and 3 days post-infection (*n* = 4–6, ****P < 0.0001).

Following our observation that IFN-γ-stimulated gene expression was dampened in the absence of TNF-α, we hypothesized that there must be a perturbation of IFN-γ signaling at either the receptor level or the downstream signaling mechanism leading to transcription (Fig. 2A). We investigated IFN-γ receptor 1 (IFNGR1) and IFNGR2 to determine if there were any alterations in lung cell surface receptor expression due to the absence of TNF-α. We previously observed that at 3 dpi, cells most significantly impacted by infection were CD64^+^, while the CD64^−^ infected populations displayed no significant differences (Fig. 4A and B). Though there were minor differences, we found that CD45^+^ CD64^+^ lung cells were comparable to CD45^+^ F4/80^+^ lung cells (Fig. S2). At 2 dpi, we observed a marked decrease in IFNGR1 in CD64^+^ and CD64^−^ leukocytes in response to infection, followed by a sharp restoration in these receptors at 3 dpi compared to uninfected controls (Fig. 5A and B). IFNGR1 expression was slightly diminished in the absence of TNF-α in CD45^+^ CD64^+^ cells at 3 dpi, but this change was not observed in CD45^+^ CD64^−^ cells (Fig. 5C and D).

### Alterations in pSTAT1 during *H. capsulatum* infection

Since the absence of TNF-α was not accompanied by diminished expression of IFNGR1, we asked if TNF-α blockade altered STAT1 signaling in macrophages. In control macrophages, there was a progressive increase in the expression of pSTAT1 (Ser727) from 1 to 2 dpi. In contrast, that increase was not evident in recipients of anti-TNF-α. Consequently, by 2 dpi, striking differences emerged between infected controls and those receiving anti-TNF-α. We found a significant decrease in pSTAT1 (Ser727) in mice that received anti-TNF-α at 2 dpi in CD45^+^ CD64^+^ cells, with far fewer cells detected as pSTAT1^+^ at 2 dpi compared to 1 dpi (Fig. 5F and G). By 3 dpi, however, pSTAT1 (Ser727) expression was markedly lower than at 2 dpi in both treatment groups and no longer differed between control and anti-TNF-α-treated mice. In contrast, the number of pSTAT1+ cells increased in both groups at 3 dpi.

## DISCUSSION

In this study, we investigated the interaction between TNF-α and IFN-γ in the innate immune response to *H. capsulatum*. Though required separately, together the two cytokines produce a robust early response against *H. capsulatum* replication. However, in the absence of TNF-α, macrophages were unable to impede replication of yeasts when stimulated with exogenous IFN-γ. Transcriptional studies revealed impaired expression of IFN-γ-stimulated genes associated with primary macrophage activation in the absence of TNF-α. Neither metabolic nor reactive nitrogen species-related stress explained the dampened antimicrobial macrophage stimulation. We discovered that TNF-α was requisite for STAT1 signaling induced by IFN-γ during infection-induced downregulation of IFNGR1. Thus, the ability of macrophages to restrain fungal growth relies on TNF-α enabling signaling by IFN-γ.

To control infection with *H. capsulatum* optimally, macrophages must be activated by IFN-γ. Otherwise, intracellular yeast cells continue to replicate. Paradoxically, TNF-α is also necessary for clearance of the fungus even though it does not directly arm macrophages to kill the fungus (25). Our data reveal that the two cytokines are interdependent in an unexpected manner. Without either cytokine, host cells are unable to control infection during innate immunity, and the bridge between innate and adaptive immunity is severed. One likely explanation for the necessity of TNF-α is that its absence reduces IFN-γ production, but this is not the case (14). Our findings raised the possibility that interdependence exists between the two cytokines to properly stimulate an immune response against infection. To explore this, we analyzed several properties of macrophage function. The absence of TNF-α did not alter the metabolism of IFN-γ-stimulated macrophages nor did it alter production of NO despite dampening expression of *Nos2*. The unanticipated result was that IFN-γ signaling was perturbed in the absence of TNF-α.

We demonstrated that decreased immunity caused by a loss of either cytokine occurs 3 days after exposure to *H. capsulatum* as lung cells are unable to restrict yeast replication. Prior work had focused principally on the course of infection during the adaptive phase (14). We found that both cytokines are critically important for control of infection prior to the onset of adaptive immunity. We noted that TNF-α altered host susceptibility to infection. TNF-α blockade increased the number of AMs, IMs, and monocytes infected compared to the control but not the number of yeasts per macrophage. Since IFN-γ was important early in infection, the results suggest that an innate lymphocyte, such as natural killer cells or type 1 innate lymphocyte cells, are triggered to produce this cytokine similarly to the immune response to other like pathogens (26–29). This contrasts with the role of CD4^+^ cells during the adaptive phase as primary producers of IFN-γ (30).

IFNGR1 is the ligand-binding chain of the dimeric IFNGR. It is the critical receptor responsible for transducing signals (31). Expression of IFNGR1 was diminished on macrophages from infected mice when compared to uninfected mice at 2 dpi. Reduced IFNGR1 has been observed in macrophages from patients infected with *Mycobacterium tuberculosis* infection and in a human macrophage line infected with this bacterium. Because IFN-γ is a key element in the clearance of *M. tuberculosis*, the data have been interpreted to suggest that the reduction in IFNGR1 potentially reduces IFN-γ signaling, thus limiting the ability of macrophages to become armed (5, 32). Hence, a diminution in the expression of IFNGR1 on the principal effector host cells by *H. capsulatum* or *M. tuberculosis* may constrain the effect of IFN-γ. Consequently, these intracellular pathogens are able to survive in host cells (32). TNF-α alone has not been observed to alter the expression of IFNGR1, and the absence of TNF-α did not alter the profile of IFNGRs during *H. capsulatum* infection at 3 dpi (31). The slight decline in IFNGR1 is attributable to infection but not the presence or absence of TNF-α. One open question is whether the change is strictly confined to infected cells. However, these cells constitute a small minority of the macrophage population (0.02%–0.04% of cells); hence, the decrease in IFNGR1 is quite likely a result of soluble mediators by host cells released during the course of infection.

In the absence of TNF-α, there is a heightened number of infected AMs and IMs at 3 dpi, but the yeast burden per cell remains the same. These results indicate that inhibiting TNF-α does not change the intracellular growth rate of the fungus but rather makes resident macrophages more permissive for invasion. Parallel to diminished IFNGR1 from infection at 2 dpi, pSTAT1 (Ser727) was significantly decreased in TNF-α blockade compared to isotype control. Though TNF-α does not upregulate nor downregulate IFNGR1, the cytokine is required for optimal STAT1 signaling (31). This suggests that TNF-α functions as a compensatory signal to decreased IFN-γ signaling that preserves the ability of macrophages to exert antifungal activity on *H. capsulatum* yeasts. The fact that differences do not appear until day 2 suggests that a complex series of signaling events must transpire to sustain pSTAT1 expression in response to decreased receptor availability. Interestingly, at day 3, pSTAT1 (Ser727) expression was reduced in both treatment groups, whereas the number of pSTAT1^+^ cells increased dramatically. These findings suggest that the requirement for TNF-α in STAT1 signaling is most pronounced during the early phase of innate immunity rather than throughout the entirety of the innate immune response. Taken together, these observations support a model in which TNF-α facilitates the establishment of effective STAT1-dependent antifungal responses during a critical early window of *H. capsulatum* infection.

Although macrophage pSTAT1 (Ser727) expression was comparable between treatment groups by 3 dpi, this did not coincide with restoration of host control over fungal replication. As the immune response progresses, additional TNF-α-dependent mechanisms likely become dominant. TNF-α antagonism promotes the emergence of antigen-specific CD4^+^ CD25^+^ regulatory T cells that suppress protective immunity through an IL-10-dependent mechanism, and depletion of these cells restores host antifungal resistance (33, 34). Thus, while early TNF-α blockade impairs macrophage STAT1 activation, continued progression of infection after 3 dpi may reflect the subsequent emergence of suppressive T-cell responses rather than persistent defects in macrophage STAT1 phosphorylation alone.

Two canonical IFN-γ-regulated factors act on the JAK-STAT signaling pathway as negative feedback checkpoints. Src homology phosphatase 2 (SHP2) is a TNF-α/IFN-γ-coregulated phosphatase that is a negative STAT1 regulator but requires TNF-α for its activity as a phosphatase. This result suggests that SHP2 is unlikely to be involved in the failure of macrophages to upregulate pSTAT1 (35). An inverse case can be made for suppressor of cytokine signaling 1 (SOCS1), a canonical negative regulator of JAK-STAT signaling, as it is primarily regulated by IFN-γ but is not dependent on TNF-α for its function (36). Neither of these molecules explain the role of TNF-α in our model, nor do they explain why pSTAT1 has failed to increase.

TNF-α has been shown to enhance phosphorylation of STAT1 at both of its activation sites: tyrosine 701 and serine 727 (37). In other models, these sites are phosphorylated separately by different paths: tyrosine 701 is phosphorylated by JAKs in IFN-γ signaling, while serine 727 is phosphorylated following interactions of TNF-α in the p38 mitogen-activated protein kinase (MAPK) signaling cascade in response to stress (38, 39). If both residues must be phosphorylated for robust gene expression later when pSTAT1 acts as a transcription factor, then the p38 pathway dependence on TNF-α may explain the apparent lack of response from IFN-γ observed *in vitro* and in lung leukocytes in our study. Inhibition of p38 MAPK induced a similar repression of serine 727 phosphorylation, resulting in diminished STAT1-dependent transcription (40). When TNF-α is blocked, the p38 MAPK signaling cannot proceed, resulting in decreased phosphorylation of STAT1 at serine 727 (39, 40). These findings show that TNF-α is critical for complete, competent activation of STAT1 and indicate that if p38 MAPK activity were restored, a host infected with *H. capsulatum* may be able to recover from infection even when TNF-α has been blocked.

In summary, decoupling of TNF-α and IFN-γ by TNF-α blockade results in insufficient activation of macrophages from decreased phosphorylation of STAT1 following decreased IFN-γ recognition from diminished expression of IFNGR1. This leads to dampened expression of interferon-stimulated genes such that the cell is unable to classically activate. Consequently, though the macrophage is not more or less susceptible to *H. capsulatum* infection compared to other lung leukocytes, hosts experiencing TNF-α deficiency are unable to hinder yeast replication during innate immunity, likely due to a lack of TNF-α-mediated STAT1 phosphorylation at serine 727 that is required for sufficient expression of interferon-stimulated genes leading to macrophage activation.

## MATERIALS AND METHODS

### Mice

C57BL/6J male wild-type mice (6–12 weeks old) were obtained from Jackson Laboratory and maintained by the Department of Laboratory Animal Medicine at the University of Cincinnati for use in our studies.

### Preparation of *H. capsulatum* yeasts for *in vivo* and *in vitro* infections

The *H. capsulatum* yeast strains G217B and a tdTomato-expressing G217B (provided by Chad Rappleye of Ohio State University) were grown for 72 hours at 37°C while shaking at 200 rpm to late log/early stationary phase in F-12 medium (containing 10.6 g Ham’s F-12, 18.2 g dextrose, 1.0 g glutamic acid, 10.0 mL 100× cystine, and 6.0 g HEPES/L of distilled water). Mice were infected intranasally (i.n.) with an inoculum of 2 × 10^5^ *H. capsulatum* yeasts, while *in vitro*, macrophages were infected with a ratio of one yeast per phagocyte.

### Mouse antibody treatments and infection

Wild-type C57BL/6 mice were treated with 1 mg anti-TNF-α mAb (clone MP6-XT22, Cell Culture Company), rat IgG1 isotype control antibody (clone GL113; Leinco Technologies, Inc.), or anti-IFN-γ monoclonal antibody (clone XMG1.2; Leinco Technologies, Inc.) and its respective isotype control, rat IgG2b (clone 1-2; Leinco Technologies, Inc.). The antibodies were administered via the intraperitoneal route. After 12–24 hours, mice were infected i.n. with *H. capsulatum* yeasts.

### Lung harvest and organ culture

Mouse lungs were harvested at 1, 2, and 3 dpi and processed in a TenBroeck manual tissue homogenizer to release *H. capsulatum* yeasts. Samples were serially diluted and spread on organ culture plates (total media containing 36 g Mycosel agar, 8 g Bacto agar, 5 g dextrose, 0.1 g cystine, and 0.1% gentamicin sulfate per liter of distilled water). After 7 days, CFUs of *H. capsulatum* were counted.

### Generation of BMDMs

BMDMs were generated using mouse bone marrow suspended in 50 mL RPMI (Cytiva) containing 10% heat-inactivated fetal bovine serum (FBS, Cytiva), 0.1% gentamicin, and 0.05% 2-mercaptoethanol and placed in a culture flask. Cells were treated with recombinant mouse granulocyte-macrophage colony-stimulating factor (BioLegend) to induce differentiation of monocytes into macrophages (41). After 7 days, 0.25% trypsin (Corning) was added to the flask for 10–15 minutes and then neutralized with RPMI containing FBS to deactivate the enzyme. BMDMs were collected by gently scraping the flask. Cells were washed three times in PBS before use. Bone marrow from TNF-α^−/−^ mice was prepared identically and was generously provided by Dr. Chandrashekhar Pasare of University of Cincinnati Children’s Hospital Medical Center.

### Infection of BMDMs and recovery of yeasts

BMDMs were seeded in a 96-well flat-bottom plate to a density of 2 × 10^5^ cells per well and incubated overnight. The next day, BMDMs were infected with *H. capsulatum* yeasts and treated with 1 μg/mL anti-TNF-α mAb or rat IgG1 isotype control antibody and 10 ng/mL mouse recombinant IFN-γ (BioLegend) at the time of infection. At 2, 24, and 48 hours post-infection, BMDMs were lysed and serially diluted. After 7 days, CFUs of *H. capsulatum* were counted.

### RNA isolation, cDNA synthesis, and quantitative real-time PCR from BMDMs

The PureLink RNA Mini Kit (12183018A and 12183025; Life Technologies, including lysis buffer, 100015634; wash buffer I, 100015635; wash buffer II, 100015636; and RNase-free water, 100015,37) was used for RNA isolation from BMDMs at 6 and 24 hours post-infection. Oligo(dT)-primed cDNA was prepared by reverse transcription using, per reaction, 6 μL magnesium chloride, 3 μL 10× reverse transcriptase buffer, 3 μL deoxynucleotide triphosphate mix, 1.5 μL oligonucleotide deoxythymidine (Oligo DT) primer, 0.9 μL RNA transcriptase, 0.75 μL RNasin, and 7.35 μL ddH_2_O (RNase/DNase free). The conditions for amplification on the MiniAmp Plus Thermal Cycler (Thermo Fisher) were 42°C for 1 hour and 99°C for 5 minutes, followed by a 4°C hold. Quantitative real-time PCR analysis was performed on the Applied Biosystems 7500 Fast Real-Time PCR System (Thermo Fisher) using Taqman Fast Universal PCR Master Mix (no UNG) and Taqman gene expression assay primers (Thermo Fisher) specific for *Nos2* (mm00440502_m1), *Cxcl9* (mm00434946_m1), *Fpr2* (mm00484464_s1), *Il12b* (mm99999067_m1), *Arg1* (mm00475988_m1), *Chil3* (mm00657889_mH), and *Socs2* (mm00850544_g1). The conditions for amplification and analysis were 95°C for 20 min, followed by 40 cycles of 95°C for 3 min each and 60°C for 30 min. Gene expression was calculated according to the relative quantification method (2^−ΔΔCT^). Samples were analyzed in triplicate and normalized to the expression of internal housekeeping gene *Actb* (mm00607939_s1).

### Characterization of metabolic phenotype by extracellular flux assay

For glycolytic and mitochondrial respiration stress assays, BMDMs were seeded with 8 × 10^4^ cells in 180 μL complete RPMI per well in Seahorse XF 96-well culture microplates (Agilent) and rested overnight at 37°C in 5% CO_2_. BMDMs were infected with *H. capsulatum* and treated with rat IgG1, anti-TNF-α, or IFN-γ. After 24 hours, the cell culture medium was replaced with 180 μL Seahorse XF Dulbecco’s modified Eagle medium containing 10 mM glucose (both assays), 2 mM L-glutamine (mitochondrial stress assay only), and 1 mM sodium pyruvate (mitochondrial stress assay only) by 1-hour incubation at 37°C in ambient CO_2_. Glycolytic stress was measured as extracellular acidification rate with a Seahorse XF glycolytic stress assay kit using a Seahorse XF 96e analyzer (Agilent). Mitochondrial stress was measured via the Seahorse XF Cell Mito stress kit using a Seahorse XF96e analyzer (Agilent) and reported as oxygen consumption rate.

### Examination of nitrite concentration in BMDM supernatants

Nitrite concentration was determined using a Griess reagent nitrite measurement kit (item 13547S, Cell Signaling Technology). BMDMs were seeded 2 × 10^5^ cells/well in a 96-well plate and infected with an equal number of *H. capsulatum* yeasts to a 200 μL total per kit manufacturer’s recommendation. At 24 hours post-infection, supernatants were recovered from wells for testing. Samples were stored at −80°C until use. Nitrite concentrations for samples were calculated from absorbance measurements taken at 550 nm relative to a standard curve constructed using the nitrite standard provided in the reagent kit.

### Flow cytometry

Mice treated with anti-TNF-α or isotype control antibody, as stated previously, were infected i.n. with tdTomato-expressing G217B yeasts. At 3 days post-infection, the mice were euthanized, and their lungs were harvested for digestion in gentleMACS C-tubes (Miltenyi Biotec) containing RPMI (Cytiva) with collagenase D (item 11088882001, Roche Diagnostics) and DNase I (item 04716728001, Roche Diagnostics). Homogenates were filtered and treated with a 1× ACK lysis buffer (1.5 M NH_4_Cl, 100 mM KHCO_3_, and 1 mM EDTA in ddH_2_O, pH 7.2–7.5) for 1–3 minutes to lyse red blood cells. The remaining cells after washing were resuspended in FACS buffer (containing 2 mM NaN_3_, 2 mM EDTA, and 2% heat-inactivated FBS in 1× PBS) and counted with trypan blue.

Cells were incubated with 50 μg/mL of antimouse CD16/CD32 (C381, Leinco Technologies, Inc.) for 10 minutes and exposed to a cocktail of fluorescent monoclonal antibodies (BioLegend) containing CD45 (1:400, BV785, antimouse, Clone 30-F11, cat. 103149), F4/80 (1:400, PE-Cy5, Clone BM8, cat. 123112), CD64 (1:200, BV605, Clone X54-5/7.1, cat. 139323), CD11b (1:100, APC-Cy7, Clone M1/70, cat. 101226), CD11c (1:50, APC, Clone N418, cat. 117310), MHCII (1:200, BV711, Clone M5/114.15.2, cat. 107643), CX_3_CR_1_ (1:400, BV650, Clone SA011F11, cat. 149033), and SiglecF (1:100, BV421, Clone S17007L, cat. 155509) for 30 minutes at 4°C, then stained for viability with a Zombie UV-excitable fixable viability dye (1:100, part 77474; BioLegend) for 10 minutes at 4°C. When staining for IFNGRs, previous surface stains for CD45 and viability were used while adding antibodies for IFNGR1 (CD119, BD Optibuild, BV 605, 1:200, Clone GR20, cat. 745111) and IFNGR2 (R&D Systems, Alexa Fluor 488, 1:200, Clone MOB47, cat. FAB773G), as well as a new fluorophore for CD64 (BioLegend, PE-Cy7, 1:200, Clone X54-5/7.1, cat. 139314). All samples were fixed with 2% paraformaldehyde (PFA, J19943-K2; Thermo Scientific) for 10 minutes to ensure fixation of leukocytes and live yeasts. Between each stain and fixation, cells were washed once with FACS Buffer.

In intracellular staining, cells were fixed first with 0.4% PFA, stained for viability, then fixed again with 2% PFA. Cells were permeabilized following surface staining with 90% methanol for 10 minutes. This was followed by staining with an antibody for pSTAT1 (BioLegend; Ser727, Alexa Fluor 647, 1:100, Clone A15158B, cat. 686412, Isotype Mouse IgG1) and a requisite isotype control antibody (BioLegend; Alexa Fluor 647, 1:100, Clone MOPC-21, cat. 400136, Isotype Mouse IgG1) for purposes of comparison.

Flow cytometry was conducted on the Cytek Aurora (serial #U0294), provided by the Cincinnati Children’s Research Flow Cytometry Facility, through the acquisition software SpectroFlo. Live unmixing was performed using unstained cells, a sample of tdTomato-expressing *H. capsulatum* (fixed), and assorted single-stained antibody bead controls. ArC reactive beads (positive and negative, A10346; Invitrogen) were used for the Zombie UV fixable viability dye bead control, while UltraComp eBeads (01-2222-42, Invitrogen) were used for all other antibodies. Single-stained and fluorescence minus one controls were made for every experiment for accurate gating and analysis. Infected phagocytes were detected using a tdTomato red-expressing strain of *H. capsulatum* yeast cells. FlowJo software was used for data analysis, with the FlowClean plugin used to filter the data.

### Statistics

Data were analyzed by Student’s *t*-test when comparing only two groups. One- and two-way ANOVAs were used when data in three or more groups achieved normality, and mixed effect analyses were used when the data did not achieve normality.

## Data Availability

All data will be made available upon reasonable request.

## SUPPLEMENTAL MATERIAL

Fig. S1 iai.00302-26-s0001.tif Flow plot layouts-Gating scheme.

Fig. S2 iai.00302-26-s0002.tif Comparability of CD64 and F4/80.

Supplemental material iai.00302-26-s0003.docx Supplemental figure legends.

ASM does not own the copyrights to Supplemental Material that may be linked to, or accessed through, an article. The authors have granted ASM a non-exclusive, world-wide license to publish the Supplemental Material files. Please contact the corresponding author directly for reuse.

## References

[1] Wajant H, Siegmund D. 2019. TNFR1 and TNFR2 in the control of the life and death balance of macrophages. Front Cell Dev Biol 7:91. doi:10.3389/fcell.2019.00091 PMC6548990 31192209

[2] Jang D-I, Lee A-H, Shin H-Y, Song H-R, Park J-H, Kang T-B, Lee S-R, Yang S-H. 2021. The role of tumor necrosis factor alpha (TNF-α) in autoimmune disease and current TNF-α inhibitors in therapeutics. Int J Mol Sci 22:2719. doi:10.3390/ijms22052719 PMC7962638 33800290

[3] Boehm U, Klamp T, Groot M, Howard JC. 1997. Cellular responses to interferon-γ. Annu Rev Immunol 15:749–795. doi:10.1146/annurev.immunol.15.1.749 9143706

[4] Nagasawa M, Spits H, Ros XR. 2018. Innate lymphoid cells (ILCs): cytokine hubs regulating immunity and tissue homeostasis. Cold Spring Harb Perspect Biol 10:a030304. doi:10.1101/cshperspect.a030304 PMC6280706 29229782

[5] Kak G, Raza M, Tiwari BK. 2018. Interferon-gamma (IFN-γ): exploring its implications in infectious diseases. Biomol Concepts 9:64–79. doi:10.1515/bmc-2018-0007 29856726

[6] Nel I, Bertrand L, Toubal A, Lehuen A. 2021. MAIT cells, guardians of skin and mucosa? Mucosal Immunol 14:803–814. doi:10.1038/s41385-021-00391-w PMC7983967 33753874

[7] Tweedle JL, Deepe GS. 2018. Tumor necrosis factor alpha antagonism reveals a gut/lung axis that amplifies regulatory T cells in a pulmonary fungal infection. Infect Immun 86:1–14. doi:10.1128/IAI.00109-18 PMC5964519 29581197

[8] Tsiodras S, Samonis G, Boumpas DT, Kontoyiannis DP. 2008. Fungal infections complicating tumor necrosis factor α blockade therapy. Mayo Clin Proc 83:181–194. doi:10.4065/83.2.181 18241628

[9] Iwasaki T, Watanabe R, Ito H, Fujii T, Okuma K, Oku T, Hirayama Y, Ohmura K, Murata K, Murakami K, Yoshitomi H, Tanaka M, Matsuda S, Matsuda F, Morinobu A, Hashimoto M. 2022. Dynamics of type I and type II interferon signature determines responsiveness to anti-TNF therapy in rheumatoid arthritis. Front Immunol 13:901437. doi:10.3389/fimmu.2022.901437 PMC9208293 35734167

[10] Bosch-Nicolau P, Ubals M, Salvador F, Sánchez-Montalvá A, Aparicio G, Erra A, Martinez de Salazar P, Sulleiro E, Molina I. 2019. Leishmaniasis and tumor necrosis factor alpha antagonists in the Mediterranean basin. A switch in clinical expression. PLoS Negl Trop Dis 13:e0007708. doi:10.1371/journal.pntd.0007708 PMC6742442 31469834

[11] Kim SY, Solomon DH. 2010. Tumor necrosis factor blockade and the risk of viral infection. Nat Rev Rheumatol 6:165–174. doi:10.1038/nrrheum.2009.279 PMC3155180 20142812

[12] Candel FJ, Peñuelas M, Tabares C, Garcia-Vidal C, Matesanz M, Salavert M, Rivas P, Pemán J. 2020. Fungal infections following treatment with monoclonal antibodies and other immunomodulatory therapies. Rev Iberoam Micol 37:5–16. doi:10.1016/j.riam.2019.09.001 31843275

[13] van Loo G, Bertrand MJM. 2023. Death by TNF: a road to inflammation. Nat Rev Immunol 23:289–303. doi:10.1038/s41577-022-00792-3 PMC9665039 36380021

[14] Allendoerfer R, Deepe GS. 1998. Blockade of endogenous TNF-alpha exacerbates primary and secondary pulmonary histoplasmosis by differential mechanisms. J Immunol 160:6072–6082. doi:10.4049/jimmunol.160.12.6072 9637524

[15] Wood KL, Hage CA, Knox KS, Kleiman MB, Sannuti A, Day RB, Wheat LJ, Twigg HL III. 2003. Histoplasmosis after treatment with anti–tumor necrosis factor-α therapy. Am J Respir Crit Care Med 167:1279–1282. doi:10.1164/rccm.200206-563OC 12615627

[16] Salim T, Sershen CL, May EE. 2016. Investigating the role of TNF-α and IFN-γ activation on the dynamics of iNOS gene expression in LPS stimulated macrophages. PLoS One 11:e0153289. doi:10.1371/journal.pone.0153289 PMC4898755 27276061

[17] Allendoerfer R, Deepe GS. 1997. Intrapulmonary response to *Histoplasma capsulatum* in gamma interferon knockout mice. Infect Immun 65:2564–2569. doi:10.1128/iai.65.7.2564-2569.1997 PMC175362 9199420

[18] Wesemann DR, Benveniste EN. 2003. STAT-1α and IFN-γ as modulators of TNF-α signaling in macrophages: regulation and functional implications of the TNF receptor 1:STAT-1α complex. J Immunol 171:5313–5319. doi:10.4049/jimmunol.171.10.5313 14607933

[19] Newman SL, Gootee L. 1992. Colony-stimulating factors activate human macrophages to inhibit intracellular growth of *Histoplasma capsulatum* yeasts. Infect Immun 60:4593–4597. doi:10.1128/iai.60.11.4593-4597.1992 PMC258207 1398972

[20] Kratochvill F, Neale G, Haverkamp JM, Van de Velde L-A, Smith AM, Kawauchi D, McEvoy J, Roussel MF, Dyer MA, Qualls JE, Murray PJ. 2015. TNF counterbalances the emergence of M2 tumor macrophages. Cell Rep 12:1902–1914. doi:10.1016/j.celrep.2015.08.033 PMC4581986 26365184

[21] Subramanian Vignesh K, Landero Figueroa JA, Porollo A, Divanovic S, Caruso JA, Deepe GS. 2016. IL-4 induces metallothionein 3- and SLC30A4-dependent increase in intracellular Zn^2+^ that promotes pathogen persistence in macrophages. Cell Rep 16:3232–3246. doi:10.1016/j.celrep.2016.08.057 PMC5603080 27653687

[22] Nittler MP, Hocking-Murray D, Foo CK, Sil A. 2005. Identification of *Histoplasma capsulatum* transcripts induced in response to reactive nitrogen species. Mol Biol Cell 16:4792–4813. doi:10.1091/mbc.e05-05-0434 PMC1237084 16030248

[23] Evans HM, Schultz DF, Boiman AJ, McKell MC, Qualls JE, Deepe GS Jr. 2021. Restraint of fumarate accrual by HIF-1α preserves miR-27a-mediated limitation of interleukin 10 during infection of macrophages by *Histoplasma capsulatum*. mBio 12:e0271021. doi:10.1128/mBio.02710-21 PMC8576535 34749531

[24] Verberk SGS, de Goede KE, Gorki FS, van Dierendonck X, Argüello RJ, Van den Bossche J. 2022. An integrated toolbox to profile macrophage immunometabolism. Cell Rep Methods 2:100192. doi:10.1016/j.crmeth.2022.100192 PMC9046227 35497494

[25] Leenen PJ, Canono BP, Drevets DA, Voerman JS, Campbell PA. 1994. TNF-α and IFN-γ stimulate a macrophage precursor cell line to kill *Listeria monocytogenes* in a nitric oxide-independent manner. J Immunol 153:5141–5147. doi:10.4049/jimmunol.153.11.5141 7525724

[26] Thäle C, Kiderlen AF. 2005. Sources of interferon-gamma (IFN-γ) in early immune response to *Listeria monocytogenes*. Immunobiology 210:673–683. doi:10.1016/j.imbio.2005.07.003 16323704

[27] Alspach E, Lussier DM, Schreiber RD. 2019. Interferon γ and its important roles in promoting and inhibiting spontaneous and therapeutic cancer immunity. Cold Spring Harb Perspect Biol 11:a028480. doi:10.1101/cshperspect.a028480 PMC6396335 29661791

[28] Feng E, Monteiro JK, Portillo AL, Balint E, Ashkar AA. 2023. Natural killer cell-derived interferon-γ regulates macrophage-mediated immunopathology during viral infection. J Infect Dis 228:834–839. doi:10.1093/infdis/jiad084 PMC10547451 36994782

[29] Wesley JD, Robbins SH, Sidobre S, Kronenberg M, Terrizzi S, Brossay L. 2005. Cutting edge: IFN-γ signaling to macrophages is required for optimal Vα14i NK T/NK cell cross-talk. J Immunol 174:3864–3868. doi:10.4049/jimmunol.174.7.3864 15778340

[30] Allendörfer R, Brunner GD, Deepe GS Jr. 1999. Complex requirements for nascent and memory immunity in pulmonary histoplasmosis. J Immunol 162:7389–7396. doi:10.4049/jimmunol.162.12.7389 10358191

[31] Johnson B, Panek P, Yu A, Fischer E, Koba M, Mendoza Hermosillo D, Capaldo CT. 2022. Interferon gamma upregulates the cytokine receptors IFNGR1 and TNFRSF1A in HT-29-MTX E12 cells. Cytokine 156:155892. doi:10.1016/j.cyto.2022.155892 PMC9636846 35653895

[32] Kak G, Tiwari BK, Singh Y, Natarajan K. 2020. Regulation of interferon-γ receptor (IFN-γR) expression in macrophages during *Mycobacterium tuberculosis* infection. Biomol Concepts 11:76–85. doi:10.1515/bmc-2020-0006 32271156

[33] Deepe GS, Gibbons RS. 2008. TNF-α antagonism generates a population of antigen-specific CD4^+^CD25^+^ T cells that inhibit protective immunity in murine histoplasmosis. J Immunol 180:1088–1097. doi:10.4049/jimmunol.180.2.1088 PMC2447823 18178849

[34] Valencia X, Stephens G, Goldbach-Mansky R, Wilson M, Shevach EM, Lipsky PE. 2006. TNF downmodulates the function of human CD4^+^CD25^hi^ T-regulatory cells. Blood 108:253–261. doi:10.1182/blood-2005-11-4567 PMC1895836 16537805

[35] You M, Flick LM, Yu DH, Feng GS. 2001. Modulation of the nuclear factor κB pathway by Shp-2 tyrosine phosphatase in mediating the induction of interleukin (IL)-6 by IL-1 or tumor necrosis factor. J Exp Med 193:101–110. doi:10.1084/jem.193.1.101 PMC2195877 11136824

[36] Hu X, Ivashkiv LB. 2009. Cross-regulation of signaling pathways by interferon-γ: implications for immune responses and autoimmune diseases. Immunity 31:539–550. doi:10.1016/j.immuni.2009.09.002 PMC2774226 19833085

[37] Han Q, Liu J, Zhang J, Shen Q, Zhang J, Fei S, Chen H, Sun L, Huang Z, Han Z, Tao J, Gu M, Ju X, Tan R. 2026. Dual phosphorylation of STAT1 at Y701/S727 by TNFα drives AIM2-mediated PANoptosis of renal tubular epithelial cells and fibrotic progression in renal allografts. Int J Biol Sci 22:582–600. doi:10.7150/ijbs.123441 PMC12780947 41522348

[38] Akagha MJ, Georgolopoulos G, Martin D, Meissl K, Amenitsch L, Vogl C, Farlik M, Fortelny N, Halbritter F, Müller M, Decker T, Strobl B. 2026. IFNγ shapes macrophage inflammatory responses by STAT1 isoform-specific epigenetic and transcriptional mechanisms. BMC Genomics 27:256. doi:10.1186/s12864-026-12601-5 PMC12973885 41645061

[39] Kovarik P, Stoiber D, Eyers PA, Menghini R, Neininger A, Gaestel M, Cohen P, Decker T. 1999. Stress-induced phosphorylation of STAT1 at Ser727 requires p38 mitogen-activated protein kinase whereas IFN-γ uses a different signaling pathway. Proc Natl Acad Sci USA 96:13956–13961. doi:10.1073/pnas.96.24.13956 PMC24172 10570180

[40] Goh KC, Haque SJ, Williams BR. 1999. p38 MAP kinase is required for STAT1 serine phosphorylation and transcriptional activation induced by interferons. EMBO J 18:5601–5608. doi:10.1093/emboj/18.20.5601 PMC1171628 10523304

[41] Trouplin V, Boucherit N, Gorvel L, Conti F, Mottola G, Ghigo E. 2013. Bone marrow-derived macrophage production. J Vis Exp 2013:e50966. doi:10.3791/50966 PMC3991821 24300014

