# Supplementary material for: Tumor necrosis factor-α coordinates the macrophage innate immune response to Histoplasma capsulatum
Source: Infect Immun. 2026 Sep 29;94(11):e00302-26. doi: 10.1128/iai.00302-26 (PMC13750115; doi:10.1128/iai.00302-26)
Supplement: Supplementary file 1 [file iai.00302-26-s0003.docx]

**Supplemental Figure Legends**

**Supplemental Figure 1:** **Cell surface flow cytometry gating scheme.** Representative flow cytometry panels from one independent experiment of control infected cells.

**Supplemental Figure 2: CD45^+^ CD64^+^ lung cells are comparable to CD45^+^ F4/80^+^ lung cells.** Comparison of (A) dTomato^+^ MFI and (B) dTomato^+^ cell counts in CD45^+^ CD64^+^ lung cells compared to CD45^+^ F4/80^+^ lung cells between isotype control and anti-TNF-α antibody treatments
